# Extracting speech spectrogram of speech signal based on generalized S-transform

**DOI:** 10.1371/journal.pone.0317362

**Published:** 2025-01-13

**Authors:** Li Jiashen, Zhang Xianwu

**Affiliations:** College of Computer Science and Technology, Xinjiang University, Urumqi, Xinjiang, China; Mae Fah Luang University, THAILAND

## Abstract

In speech signal processing, time-frequency analysis is commonly employed to extract the spectrogram of speech signals. While many algorithms exist to achieve this with high-quality results, they often lack the flexibility to adjust the resolution of the extracted spectrograms. However, applications such as speech recognition and speech separation frequently require spectrograms of varying resolutions. The flexibility of an algorithm in providing different resolutions is crucial for these applications. This paper introduces the generalized S-transform, and explains its fundamental theory and algorithmic implementation. By adjusting parameters, the proposed method flexibly produces spectrograms with different resolutions, offering a novel and effective approach to obtain speech signal spectrograms. The algorithm enhances the traditional Stockwell transform (S-transform) by incorporating a low-pass filtering function and introducing two adjustable parameters. These parameters modify the Gaussian window function of the basic S-transform, resulting with the generalized S-transform with customizable time-frequency resolution. Finally, this paper presents simulation experiments using both synthesized signals and real speech datas, comparing with the generalized S-transform with several commonly used spectrogram extraction algorithms. The experiments demonstrate that the generalized S-transform is feasible and effective, particularly when it is combined with the generalized fundamental frequency profile. The results indicate that this method is a viable and effective in obtaining spectrograms of speech signals, and has potential application in speech feature extraction and speech recognition. The pure speech dataset used in the experiments is sourced from a downloadable database and partially from a recorded speech set.

## 1 Introduction

As a type of non-stationary signal, speech signals are often effectively analyzed and processed using time-frequency analysis. For example, [1] presents an algorithm based on time-frequency analysis to extract the fundamental period of a speech signal. Time-frequency analysis represents a one-dimensional time signal as a two-dimensional time-frequency density function, which aims to reveal the frequency components within the signal and how these components vary over time. In speech signal processing, the visual representation that simultaneously displays time, frequency, and energy distribution is commonly referred to as a speech spectrogram [2].

Speech spectrograms, which provide a graphical display of speech feature information in both the time and frequency domains, are widely used in applications such as speech recognition and speech feature extraction. For instance, a new method for extracting features from speech spectrograms is proposed in [3]. Additionally, speech spectrograms have been combined with convolutional neural networks in [4] to improve speech intelligibility in individuals with speech impairments and serve as a tool for clinical symptom management. Common algorithms used in speech signal processing for extracting spectrograms include the Short-Time Fourier Transform (STFT) and Wavelet Transform [5]. The STFT is employed in [6–8] to extract speech spectrograms, which are then combined with deep learning for clinical diagnosis and speech quality enhancement. Wavelet transform is used in [9, 10] to extract speech spectrograms and is combined with other methods for speech enhancement research. However, STFT uses fixed-size sliding windows, which limits its ability to accurately analyze low-frequency signals with periods longer than the time window, and it offers relatively poor time-frequency resolution at high frequencies. Although the wavelet transform can adaptively reflect both low and high-frequency components, it is not closely related to the Fourier spectrum [2].

To address these limitations, R.G. Stockwell et al. proposed a new time-frequency analysis method known as the Stockwell transform (S-transform) [11–13], which combines the strengths and mitigates the weaknesses of STFT and wavelet transform. The S-transform introduces the multi-resolution analysis characteristics of the wavelet transform while maintaining a direct relationship with the Fourier spectrum. As a result, it has been widely used in the analysis of ground-penetrating radar, seismic wave data, and power systems [14–19].

For the window function in the S-transform is fixed, its application is limited. To enhance the flexibility and time-frequency resolution of the S-transform, Zhang Xianwu et al. modified the window function and proposed the generalized S-transform [19–21]. This variant has been practically applied to fields such as seismic data analysis, mechanical noise, and other signal processing areas. For example, [20] utilized the flexibility of the generalized S-transform for ground-penetrating radar layer identification applications. In this study, we leverage the ability of the generalized S-transform to flexibly adjust resolution in speech spectrograms, applying it to speech analysis. By adjusting the size of the window function through introduced parameters, we address the limitation of the fixed window function in the standard S-transform. To date, no scholars have applied the generalized S-transform to speech signal processing for obtaining speech spectrograms. This paper introduces the generalized S-transform as a time-frequency analysis method for flexible extraction of speech spectrograms. The extracted spectrogram can be used for tasks such as speech feature parameter extraction [22], endpoint detection [23], and more. With the advancement of artificial intelligence, spectrograms are increasingly used in deep learning models for speech recognition [24], music recognition [25], and related research. Extracting speech spectrograms using the generalized S-transform holds significant theoretical and practical value for speech analysis.

## 2 Rationale

The generalized S-transform, a time-frequency analysis method, was introduced earlier. Below, we will derive the computational formulas for both the S-transform and the generalized S-transform in detail.

### 2.1 S-transformation

R.G. Stockwell et al. combined the advantages of STFT and wavelet transform and changed the window function of STFT to Gaussian window function [11–14], i.e:

$$\begin{array}{c} \omega \left(t\right)=\frac{1}{a\sqrt{2\pi }}e^{-\frac{{\left(\tau -t\right)}^{2}}{2a^{2}}} \end{array}$$ (1)

where a in Eq (1) is the scale factor, and t, *τ* denotes the time. Let $\text{a}=\frac{1}{f}$, then the S-transform of h(t) is obtained as follows:

$$\begin{array}{c} S\left(\tau ,f\right)=\int_{-\infty }^{+\infty }h\left(t\right)\frac{\left|f\right|}{\sqrt{2\pi }}e^{-\frac{{\left(\tau -t\right)}^{2}f^{2}}{2}}e^{-i2\pi ft}dt \end{array}$$ (2)

where t, *τ* denotes the time and f denotes the frequency, both real numbers; Define the time window function of the S-transform as G(t, f),

$$\begin{array}{c} G\left(t,f\right)=\frac{\left|f\right|}{\sqrt{2\pi }}e^{-\frac{t^{2}f^{2}}{2}} \end{array}$$ (3)

The time window function G(t, f) of the S-transformation has to satisfy the following condition [21], i.e.

$$\begin{array}{c} \int_{-\infty }^{+\infty }G\left(t,f\right)dt=1 \end{array}$$ (4)

can be obtained under the condition that Eq (4) is satisfied:

$$\begin{array}{c} \int_{-\infty }^{+\infty }S\left(\tau ,f\right)d\tau =H\left(f\right) \end{array}$$ (5)

H(f) in Eq (5) is the Fourier transform of h(t), then h(t) is obtained from S(*τ*,f).

$$\begin{array}{c} h\left(t\right)=\int_{-\infty }^{+\infty }\left\{\int_{-\infty }^{+\infty }S\left(\tau ,f\right)d\tau \right\}e^{i2\pi ft}df \end{array}$$ (6)

In order to improve the efficiency of the calculation, the S-transform is usually implemented in the frequency domain, and the frequency domain S-positive transform is:

$$\begin{array}{c} S\left(\tau ,f\right)=\int_{-\infty }^{+\infty }H\left(\eta +f\right)e^{-\frac{2\pi^{2}\eta^{2}}{f^{2}}}e^{i2\pi \eta \tau }d\eta ,\left(f\neq 0\right) \end{array}$$ (7)

### 2.2 Generalized S-transform (GST)

The generalized S-transform (GST), as proposed in this paper, extends the traditional S-transform by incorporating a low-pass filtering function. Additionally, it introduces a regularization parameter within the time window function. This parameter plays a crucial role in adjusting both the time and frequency resolutions of the GST. By varying the magnitude of this parameter, researchers can effectively control the trade-off between time resolution and frequency resolution in the transformed signal [20, 21].

Define the time window function of the generalized S-transform as the W(t,f,λ_*G*_,λ_*L*_):

$$\begin{array}{cc} & W(t,f,\lambda_{G},\lambda_{L})=\\ & \int_{-\infty }^{+\infty }\frac{\lambda_{G}|f|}{\sqrt{2\pi }}e^{-\frac{k^{2}{\lambda_{G}}^{2}f^{2}}{2}}\frac{sin[2\pi \lambda_{L}|f|\left(t-k\right)]}{\pi (t-k)}dk,\\ & \left(\lambda_{G}>0,\lambda_{L}>0\right) \end{array}$$ (8)

In Eq (8), λ_*G*_ and λ_*L*_ represent different adjustment parameters. The parameter λ_*G*_ is used to adjust the width of the time window: the larger λ_*G*_ is, the narrower the time window becomes. This increases the temporal resolution of the generalized S transform but simultaneously reduces its frequency resolution. Conversely, the frequency resolution of the generalized S transform is adjusted by modifying the parameter λ_*L*_: the smaller λ_*L*_ is, the higher the frequency resolution becomes [20].

The time-window function of GST W(t,f,λ_*G*_,λ_*L*_) satisfies the time-window function condition for the S-transform [13].

$$\begin{array}{c} \int_{-\infty }^{+\infty }W\left(t,f,\lambda_{G},\lambda_{L}\right)dt=1 \end{array}$$ (9)

Then the GST is obtained as follows:

$$\begin{array}{c} S_{G}\left(\tau ,f,\lambda_{G},\lambda_{L}\right)=\int_{-\infty }^{+\infty }h\left(t\right)W\left(\tau -t,f,\lambda_{G},\lambda_{L}\right)e^{-i2\pi ft}dt \end{array}$$ (10)

H(f) can be obtained under the condition that Eq (9) is satisfied:

$$\begin{array}{cc} & \int_{-\infty }^{+\infty }S_{G}\left(\tau ,f,\lambda_{G},\lambda_{L}\right)d\tau \\ & =\int_{-\infty }^{+\infty }\left[\int_{-\infty }^{+\infty }h\left(t\right)W\left(\tau -t,f,\lambda_{G},\lambda_{L}\right)e^{-i2\pi ft}dt\right]d\tau \\ & =\int_{-\infty }^{+\infty }\left[\int_{-\infty }^{+\infty }W(\tau -t,f,\lambda_{G},\lambda_{L})d\tau \right]h\left(t\right)e^{-i2\pi ft}dt\\ & =H(f) \end{array}$$ (11)

H(f) in Eq (11) is the Fourier transform of the signal h(t), then the inverse transform of the GST is:

$$\begin{array}{cc} & h\left(t\right)=\int_{-\infty }^{+\infty }H\left(f\right)e^{i2\pi ft}df\\ & =\int_{-\infty }^{+\infty }\left[\int_{-\infty }^{+\infty }S_{G}\left(\tau ,f,\lambda_{G},\lambda_{L}\right)d\tau \right]e^{i2\pi ft}df \end{array}$$ (12)

In order to improve the computational efficiency, the generalized S-transform can be implemented in the frequency domain, and the frequency domain transform equation is:

$$\begin{array}{c} S_{G}\left(\tau ,f,\lambda_{G},\lambda_{L}\right)=\int_{-\infty }^{+\infty }H\left(\eta +f\right)L\left(\eta ,f\right)e^{-\frac{2\pi^{2}\eta^{2}}{\lambda_{G}^{2}f^{2}}}e^{i2\pi \eta \tau }d\eta ,\left(f\neq 0\right) \end{array}$$ (13)

In Eq (13) what L(*η*,f) is:

$$\begin{array}{c} L(\eta ,f)=\{\begin{array}{ll} 1 & \left|\eta |\leq \lambda_{L}|f\right|\\ 0 & |\eta |>\lambda_{L}|f|, \end{array} \end{array}$$ (14)

To ensure that the GST positive and inverse transformations are fully invertible, the GST is satisfied when f = 0:

$$\begin{array}{cc} & \int_{-\infty }^{+\infty }S_{G}\left(\tau ,0,\lambda_{G},\lambda_{L}\right)d\tau =H\left(0\right) \end{array}$$ (15)

## 3 Comparative analysis

The generalized S-transform enhances the original S-transform by introducing a low-pass filter and two adjustable parameters. These parameters allow for the modification of the window function size, thereby altering the resolution of the generalized S-transform. This approach combines the multi-resolution analysis capabilities of the wavelet transform while maintaining a direct relationship with the Fourier spectrum. Below, we provide a theoretical validation of the generalized S-transform and explore its application in speech signal processing.

To examine the characteristics of the generalized S-transform for analyzing time-varying signals, a time-varying signal h(t) is synthesized [20].

$$\begin{array}{c} h\left(t\right)=\{\begin{array}{ll} sin(2\pi 100t) & 0\leq t\leq 273ms\\ sin(2\pi 100t)+sin(2\pi 200t) & 274ms\leq t\leq 449ms\\ sin(2\pi 200t) & 450ms\leq t\leq 549ms\\ sin(2\pi 200t)+sin(2\pi 300t) & 550ms\leq t\leq 723ms\\ sin(2\pi 300t) & 724ms\leq t\leq 999ms, \end{array} \end{array}$$

**Fig 1:**
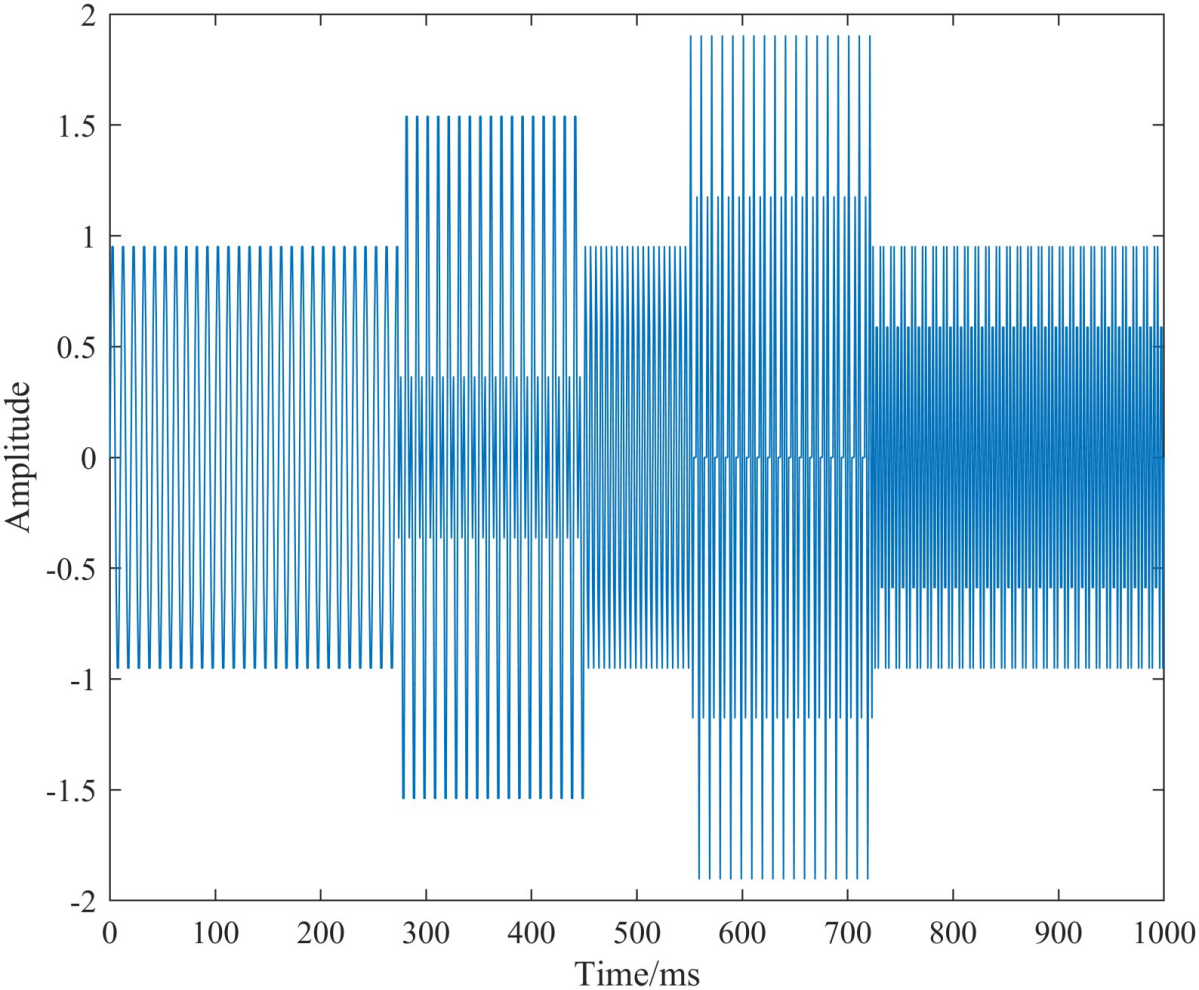
Schematic of the synthesized time-varying signal h(t) after sampling.

The signal h(t) primarily contains three frequency components: 100 Hz, 200 Hz, and 300 Hz. The starting and stopping times for each frequency component are 0 ∼ 449 ms, 274 ∼ 723 ms, and 550 ∼ 999 ms, respectively. The signal h(t) is sampled with a sampling interval of 1 ms, and the total sampling duration is 999 ms. Fig 1 presents a schematic diagram of the sampled signal h(t) [20].

### 3.1 S-transform of the synthesized signal

**Fig 2:**
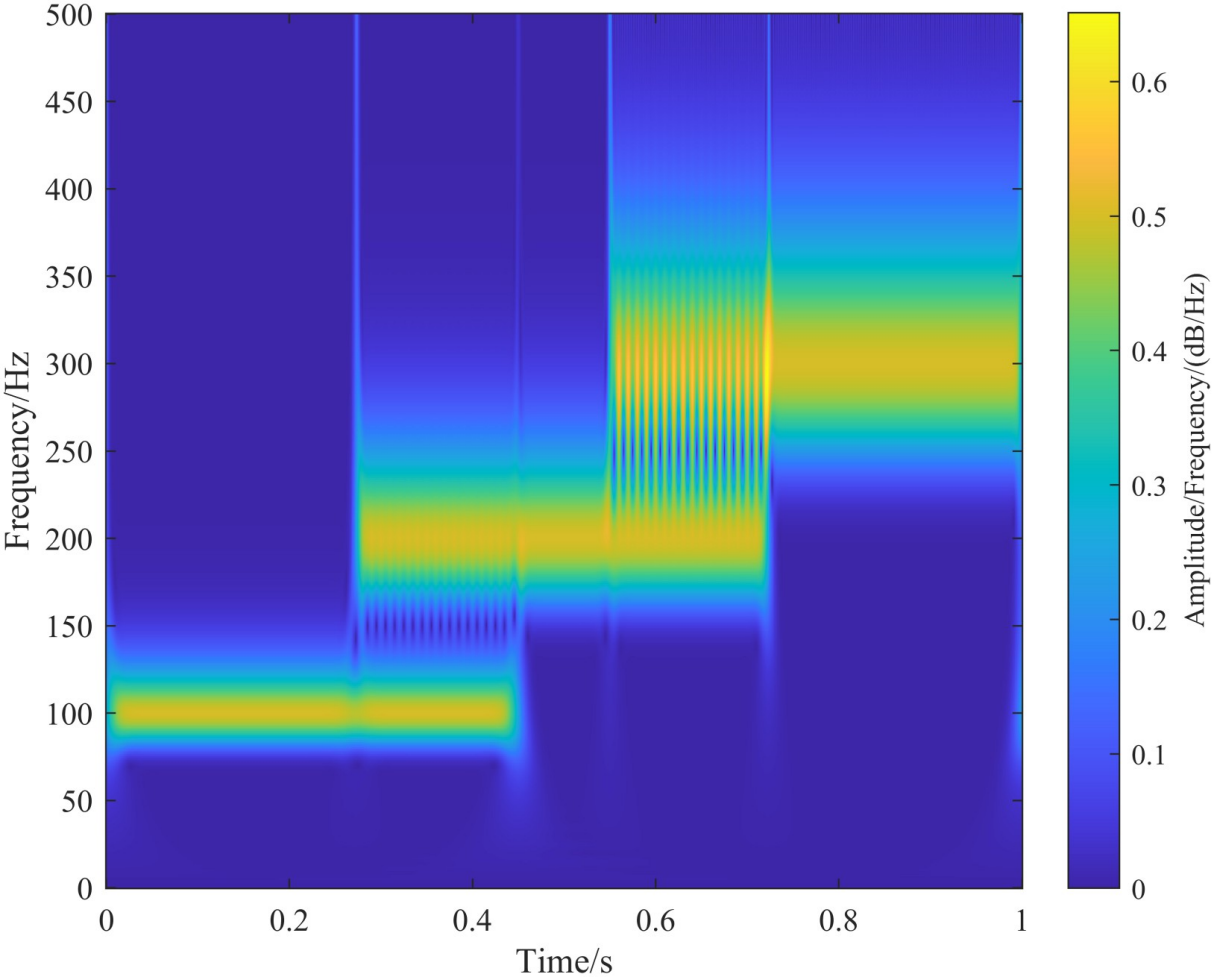
Synthesized time-varying signal S-transform results.

The S-transform is performed on the signal h(t), and the results are shown in Fig 2. In Fig 2, the frequency components of the signal and their corresponding times align with the synthesized signal h(t). As the frequency of the signal increases, the frequency resolution of the S-transform decreases, while the time resolution increases [20].

### 3.2 Generalized S-transform of synthesized signals

#### 3.2.1 Effect of the tuning parameter λ_*G*_ on the generalized S-transform

**Fig 3:**
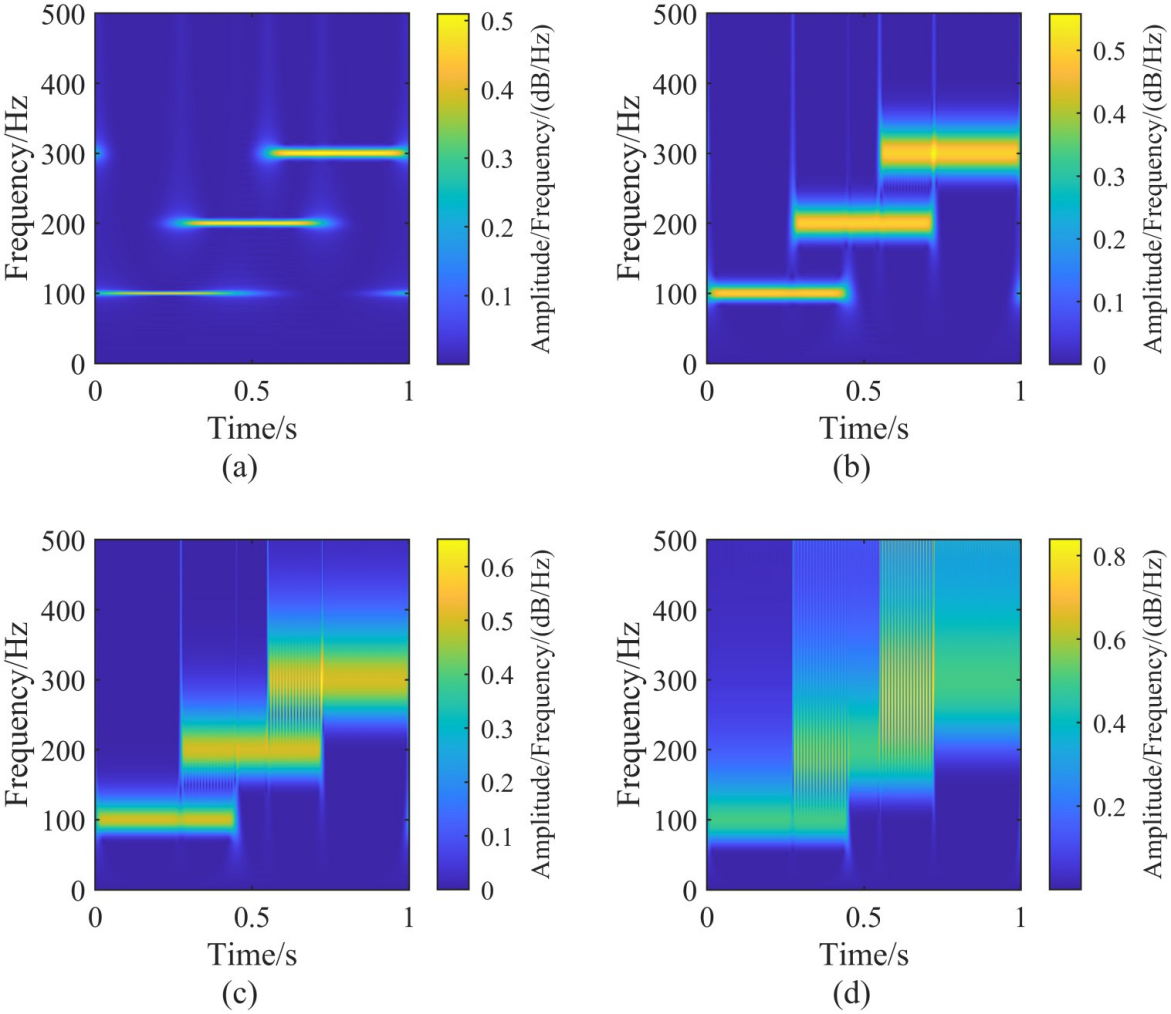
Generalized S-transform result plots of the signal h(t) corresponding to the fixed parameter λ_*L*_ = 10, adjusting the size of the parameter λ_*G*_. In the figure (a-d) are the result plots corresponding to the different parameters λ_*G*_, corresponding to the parameter sizes: (a)(λ_*G*_ = 0.1, λ_*L*_ = 10), (b)(λ_*G*_ = 0.5, λ_*L*_ = 10), (c)(λ_*G*_ = 1, λ_*L*_ = 10), (d)(λ_*G*_ = 2, λ_*L*_ = 10).

To analyze the effect of adjusting the parameter λ_*G*_ on the generalized S transform, we first fix λ_*L*_ at 10. Then, by adjusting λ_*G*_ and performing the generalized S transform on the signal h(t) for different values of λ_*G*_, we can observe the results. Fig 3 shows the impact of λ_*G*_ on the generalized S transform. From Fig 3, it can be seen that, with λ_*L*_ held constant, the time resolution of the generalized S transform increases as λ_*G*_ increases. The time resolution of the generalized S transform is higher than that of the standard S transform when λ_*G*_> 1. However, the frequency resolution of the generalized S transform decreases as λ_*G*_ increases. At λ_*G*_ = 2, due to the high time resolution and low-frequency resolution, the frequency components overlap during the periods 274 ms ∼ 449 ms and 550 ms ∼ 723 ms, making it impossible to distinguish them in the time-frequency domain after the generalized S transform [20].

#### 3.2.2 Effect of tuning parameter λ_*L*_ on the generalized S-transform

**Fig 4:**
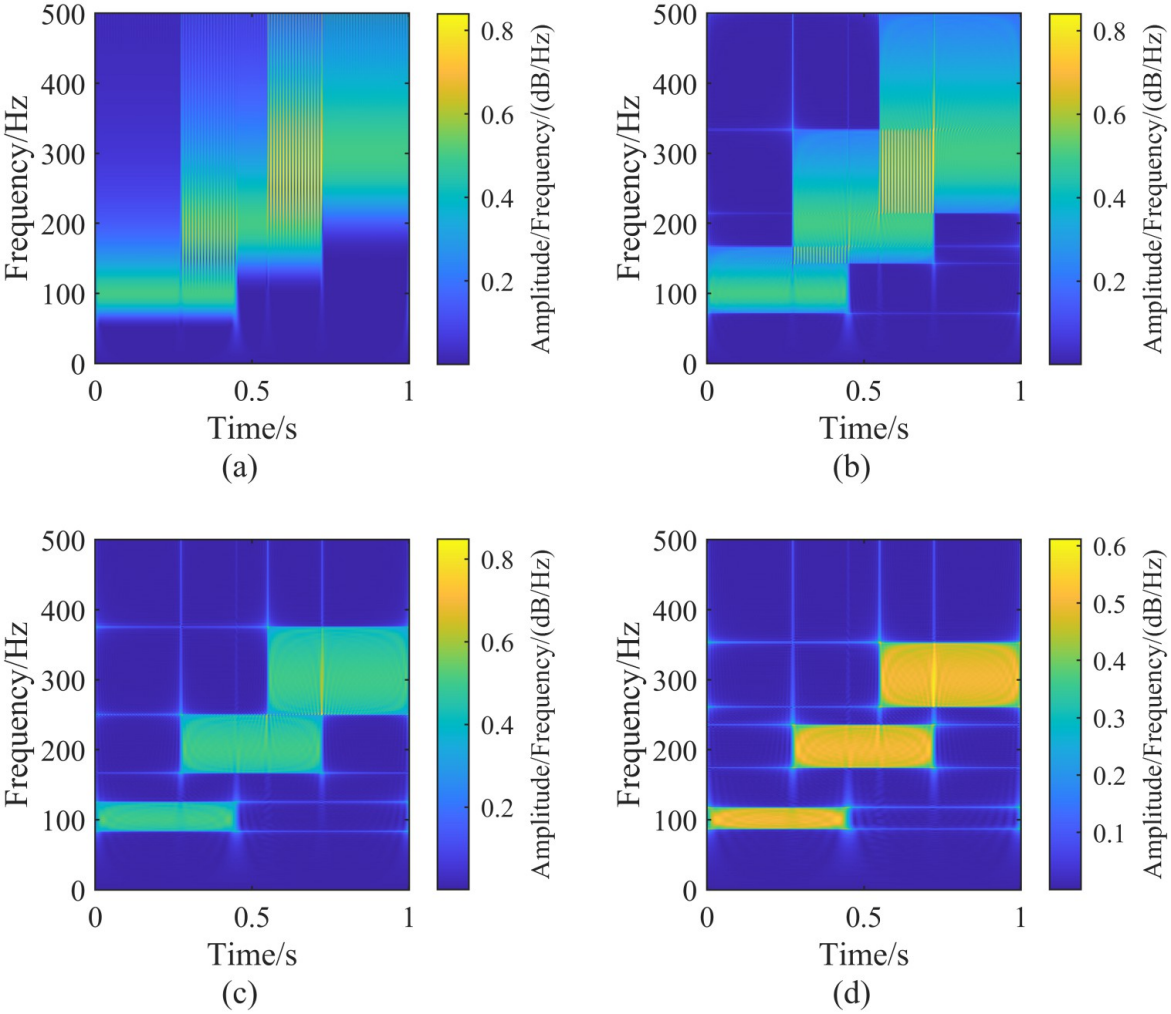
Generalized S-transform result plots of the signal h(t) corresponding to the fixed parameter λ_*G*_ = 2, adjusting the size of the parameter λ_*L*_. In the figure (a-d) are the result plots corresponding to the different parameters λ_*L*_, corresponding to the parameter sizes: (a)(λ_*G*_ = 2, λ_*L*_ = 1), (b)(λ_*G*_ = 2, λ_*L*_ = 0.4), (c)(λ_*G*_ = 2, λ_*L*_ = 0.2), (d)(λ_*G*_ = 2, λ_*L*_ = 0.15).

By fixing λ_*G*_ at 2 and adjusting λ_*L*_, we perform the generalized S-transform on the signal h(t) for different values of λ_*L*_. The results are shown in Fig 4. Comparing Figs 3d with 4a, we observe that the frequency components overlap during the periods of 274 ms ∼ 449 ms and 550 ms ∼ 723 ms. As λ_*L*_ decreases, the frequency resolution of the generalized S-transform increases, and the overlapping frequency components can be gradually separated when λ_*L*_ ≤ 0.2. When λ_*L*_ ≤ 0.2, the overlapping frequency components are separated and can be recognized in the time-frequency domain. The transform results in Figs 3d and 4a are the same, indicating that the generalized S-transform results do not change whenλ_*G*_ is increased to a certain value while λ_*L*_ remains constant. When λ_*G*_ = 1 and λ_*L*_ = +∞ the generalized S-transform and S-transform results are identical for any signal [20].

## 4 Generalized S-transform processing of speech signals

**Fig 5:**
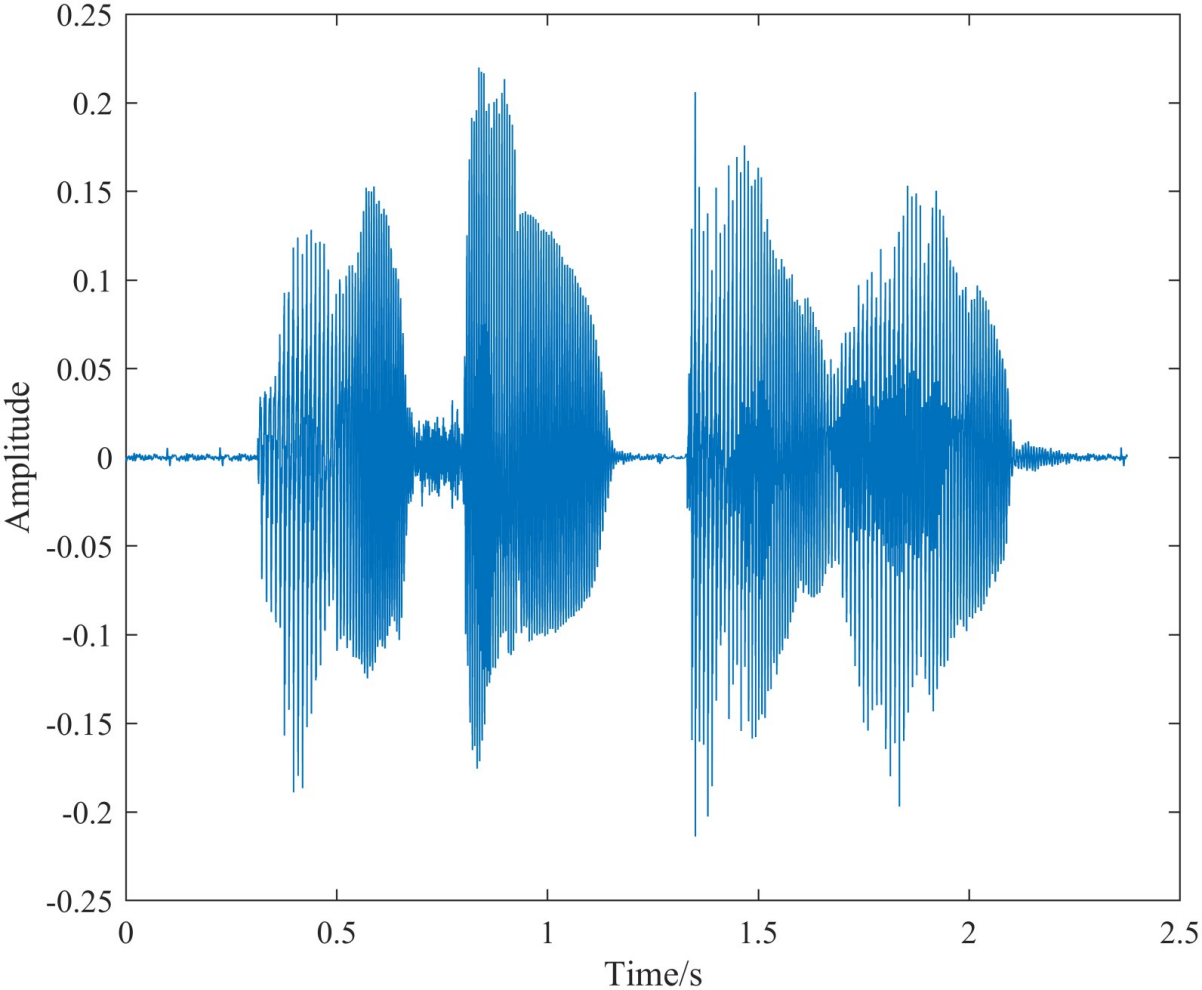
Time domain diagram of speech signal.

**Fig 6:**
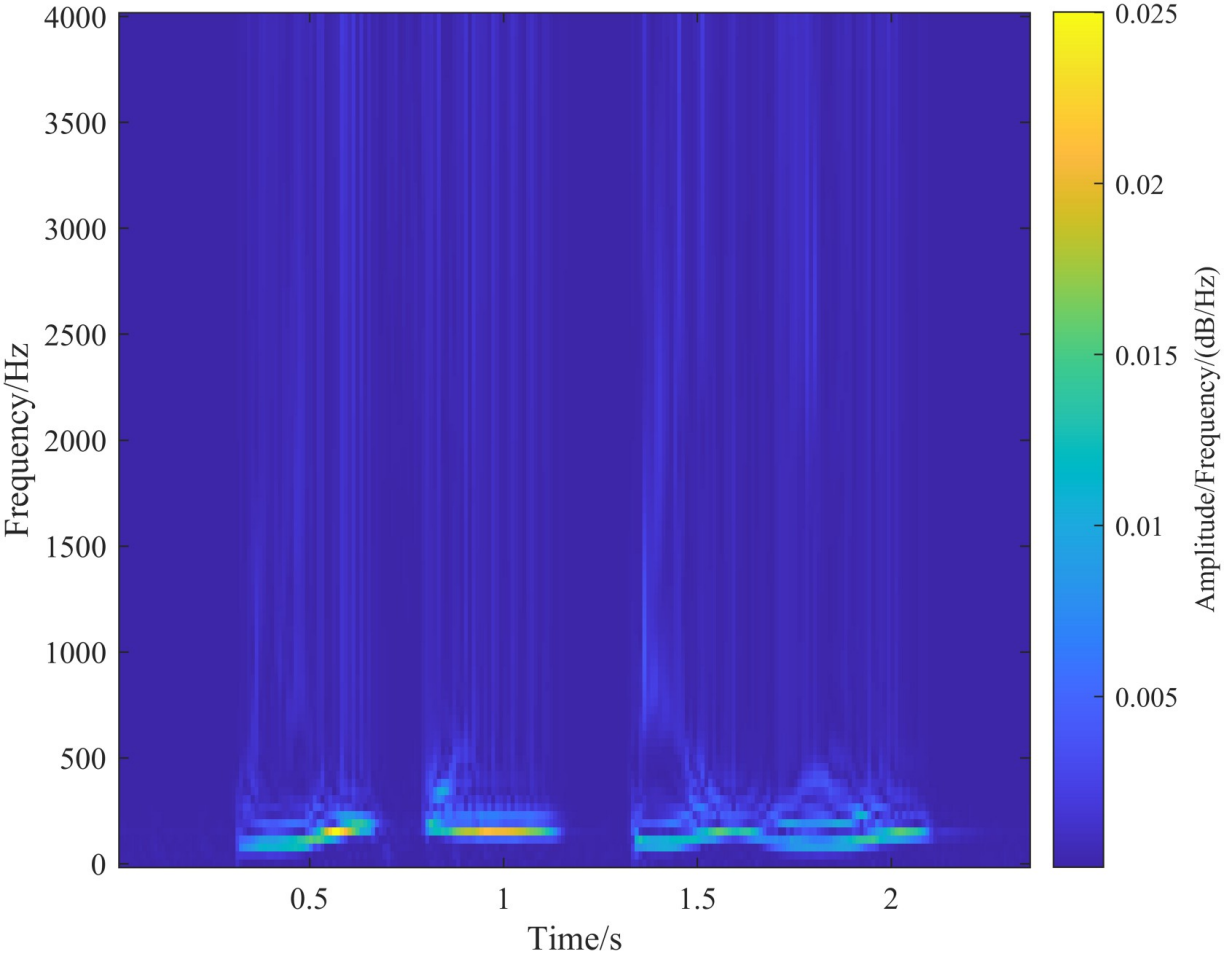
Speech signal extracted spectrogram by S-transform.

A speech signal is inherently non-stationary, but it can be considered quasi-stationary over short periods. Therefore, speech signal processing systems typically segment the signal into short-time frames of 10 ∼ 40 ms for analysis [2]. In this experiment, we used speech data from a Chinese public dataset, with a duration of approximately 2 seconds, a sampling frequency of 8000 Hz, and a single channel. The content is the phrase ‘blue sky and white clouds.’ S1 Video. We selected a frame length of 30 ms and a frame shift of 15 ms for the analysis, as shown in Fig 5. Each frame of the speech signal was processed using the S-transform and generalized S-transform. Fig 6 shows the resulting speech spectrogram extracted using the S-transform. The temporal resolution of the spectrogram is determined by the clarity of curves parallel to the vertical axis (frequency axis) and perpendicular to the horizontal axis (time axis). Similarly, the frequency resolution is determined by the clarity of curves parallel to the horizontal axis (time axis) and perpendicular to the vertical axis (frequency axis). As shown in Fig 6, the spectrogram obtained by the S-transform exhibits higher temporal resolution in the high-frequency regions and higher frequency resolution in the low-frequency regions. However, there is some frequency overlap in the low-frequency region, and the fundamental frequency and the second formant are not distinctly separated.

**Fig 7:**
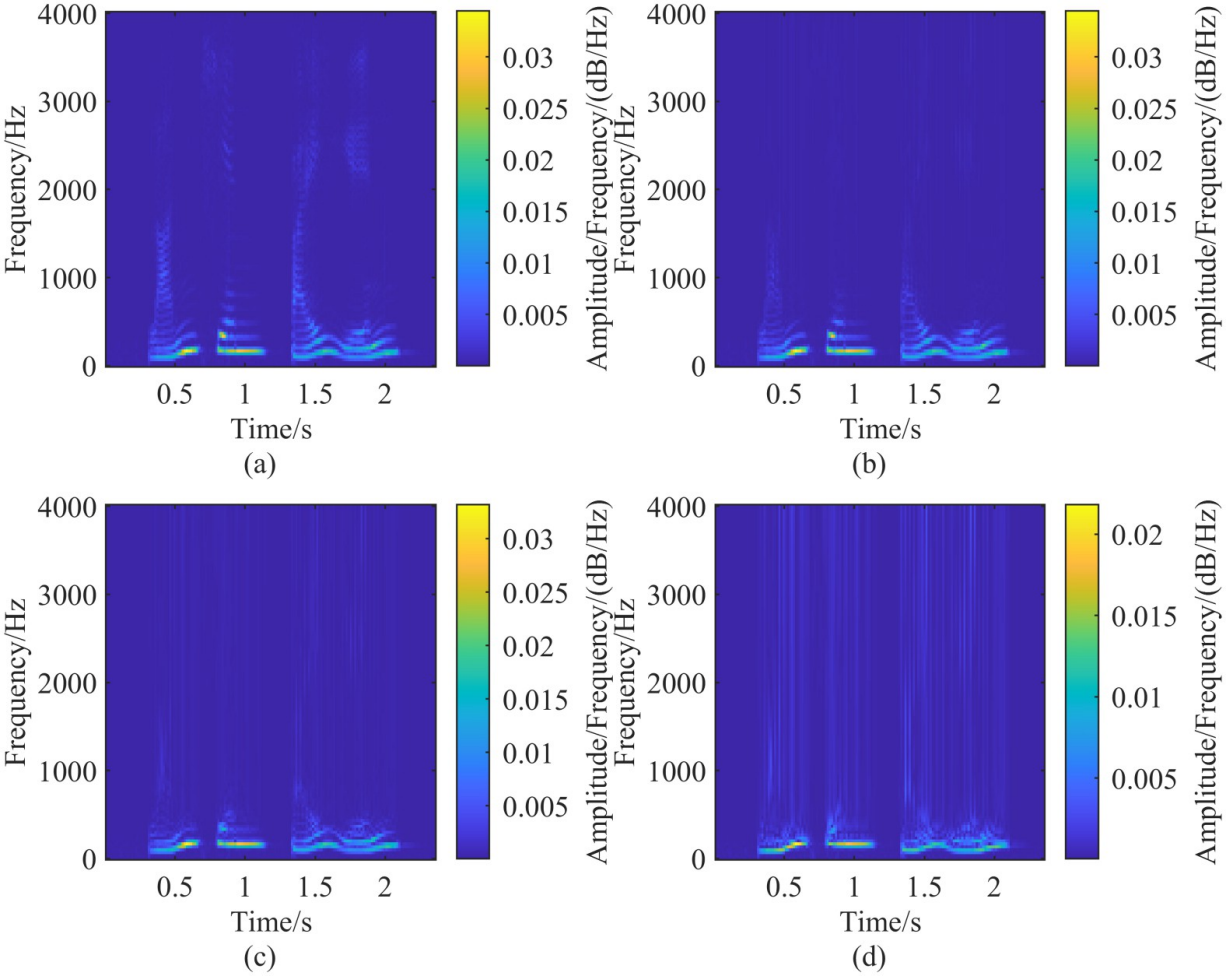
Fixing λ_*L*_ = 1 and adjusting the size of λ_*G*_, with the gradual increase of λ_*G*_, the time resolution of the high-frequency part of the spectrogram gradually increases and the frequency resolution gradually decreases. (a-d) Figures are the spectrograms extracted with different λ_*G*_ sizes, and the corresponding parameter settings are: (a)(λ_*G*_ = 0.01, λ_*L*_ = 1), (b)(λ_*G*_ = 0.25, λ_*L*_ = 1), (c)(λ_*G*_ = 0.5, λ_*L*_ = 1), (d)(λ_*G*_ = 0.95, λ_*L*_ = 1).

When applying the generalized S-transform to speech data, it is essential to select appropriate adjustment parameters λ_*G*_ and λ_*L*_. A practical approach is to first set λ_*L*_ = 1 and then adjust the parameter λ_*G*_ based on the transformation results. As discussed in the previous section, a larger λ_*G*_ increases the temporal resolution of the generalized S-transform. To adjust λ_*G*_, we can initially set it to 0.01 and then incrementally increase λ_*G*_ by 0.01 in each step, analyzing the corresponding results. Fig 7 illustrates the speech spectrograms obtained with λ_*G*_ values of 0.01, 0.25, 0.5, and 0.95, respectively. As λ_*G*_ increases, the temporal resolution of the speech spectrogram improves, while the frequency resolution decreases. This demonstrates that the temporal resolution of the spectrogram can be effectively controlled by adjusting the value of λ_*G*_.

**Fig 8:**
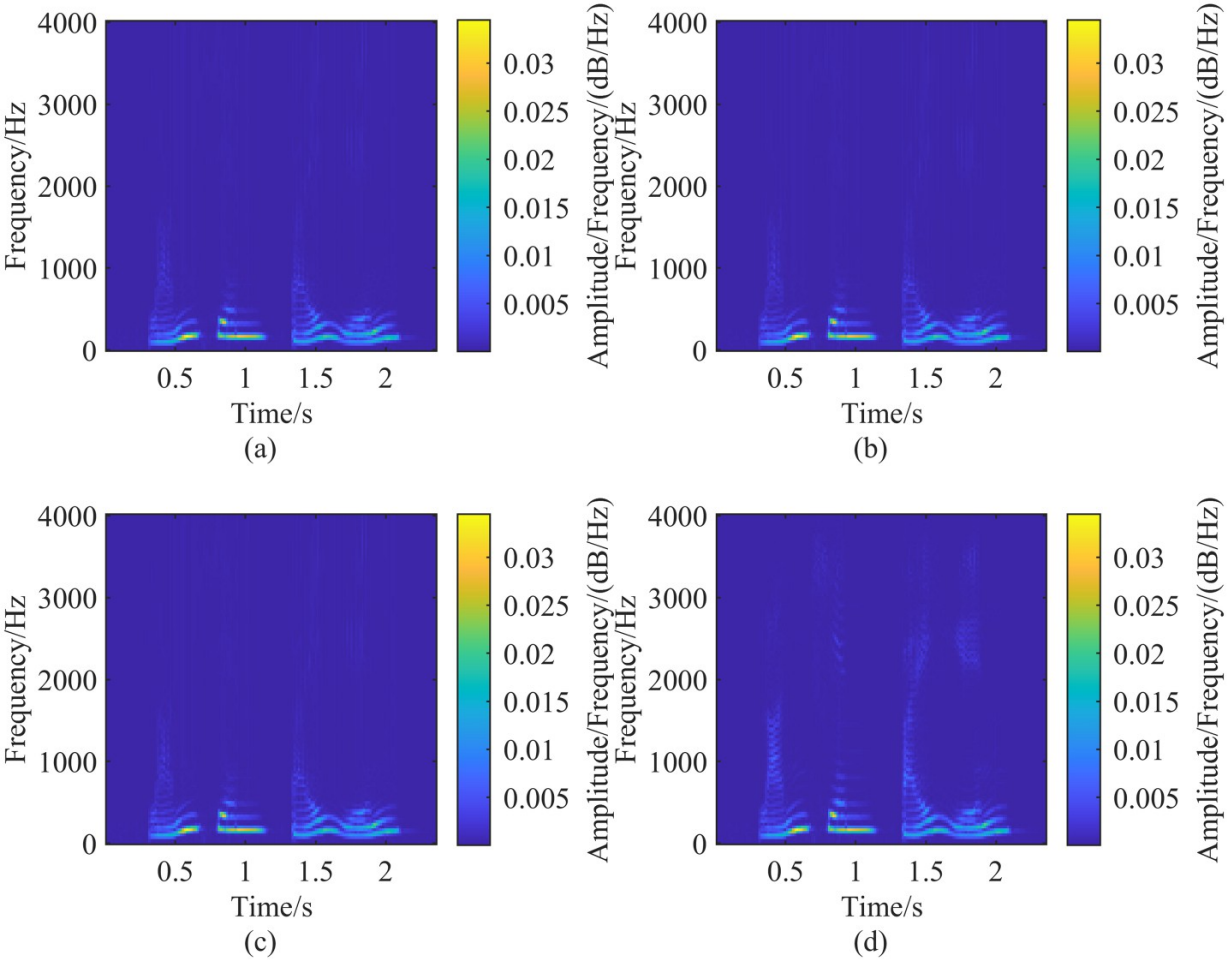
Fixing λ_*G*_ = 0.25 and adjusting the size of λ_*L*_, the time resolution of the high-frequency part of the spectrogram gradually decreases with the gradual decrease of λ_*L*_, and the frequency resolution gradually increases, (a-d) Figs. are the spectrograms extracted by different sizes of λ_*L*_, and the corresponding parameter settings are: (a)(λ_*G*_ = 0.25, λ_*L*_ = 1), (b)(λ_*G*_ = 0.25, λ_*L*_ = 0.5), (c)(λ_*G*_ = 0.25, λ_*L*_ = 0.1), (d)(λ_*G*_ = 0.25, λ_*L*_ = 0.01).

In the process of selecting λ_*G*_, increasing λ_*G*_ gradually improves time resolution but simultaneously reduces the frequency resolution of the generalized S-transform. To balance this trade-off, after determining the adjustment parameter λ_*G*_, λ_*L*_ should be reduced appropriately. The optimal values of λ_*G*_ and λ_*L*_ are selected when the different resolution components in the transform result are better separated. In the analysis of speech signals using the generalized S-transform, λ_*G*_ is set to 0.25 and λ_*L*_ to 1, then λ_*L*_ is gradually decreased in steps of 0.01. The results of the generalized S-transform for different λ_*L*_ values are compared, as shown in Fig 8, which displays the spectrograms for λ_*L*_ = 1, 0.5, 0.1, and 0.01, respectively. As λ_*L*_ decreases, the time resolution of the high-frequency portion of the spectrogram decreases while the frequency resolution increases. When λ_*G*_ = 0.25 and λ_*L*_ = 0.01, the frequency components in the spectrogram of Fig 8(d) are well separated, making it a suitable representation of the corresponding speech signal.

**Fig 9:**
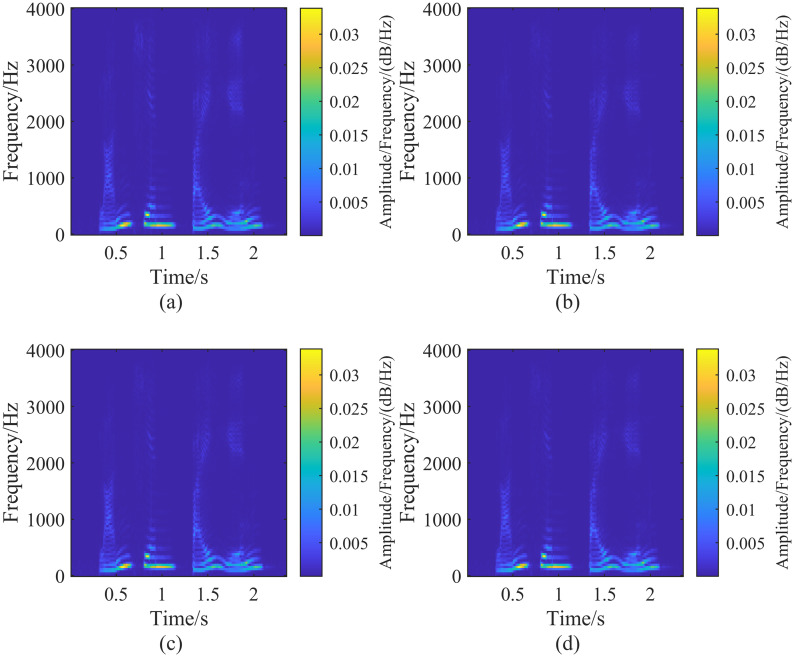
Speech spectrograms extracted by adjusting the size of λ_*L*_ corresponding to the generalized S-transform when λ_*G*_ = 0.05, and (a-d) Figs. are the speech spectrograms extracted by different λ_*L*_ sizes corresponding to the size of the parameter settings, respectively: (a)(λ_*G*_ = 0.05, λ_*L*_ = 0.01), (b)(λ_*G*_ = 0.05, λ_*L*_ = 0.1), (c)(λ_*G*_ = 0.05, λ_*L*_ = 1), (d)(λ_*G*_ = 0.05, λ_*L*_ = 10).

**Fig 10:**
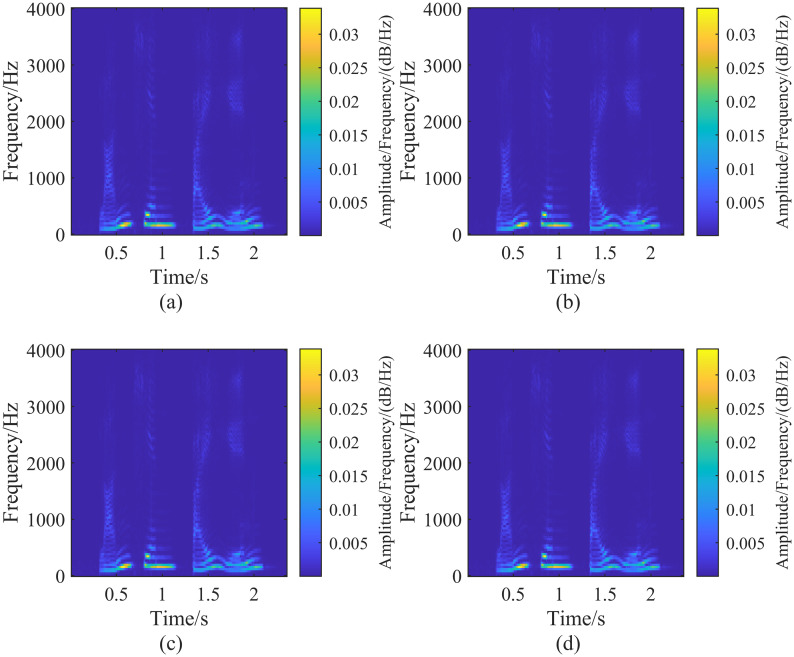
Speech spectrograms extracted by adjusting the size of corresponding to the generalized S-transform when λ_*L*_ = 0.01, and (a-d) Figs. are the speech spectrograms extracted by different λ_*G*_ sizes corresponding to the size of the parameter settings, respectively: (a)(λ_*G*_ = 0.01, λ_*L*_ = 0.01), (b)(λ_*G*_ = 0.1, λ_*L*_ = 0.01), (c)(λ_*G*_ = 1, λ_*L*_ = 0.01), (d)(λ_*G*_ = 10, λ_*L*_ = 0.01).

**Fig 11:**
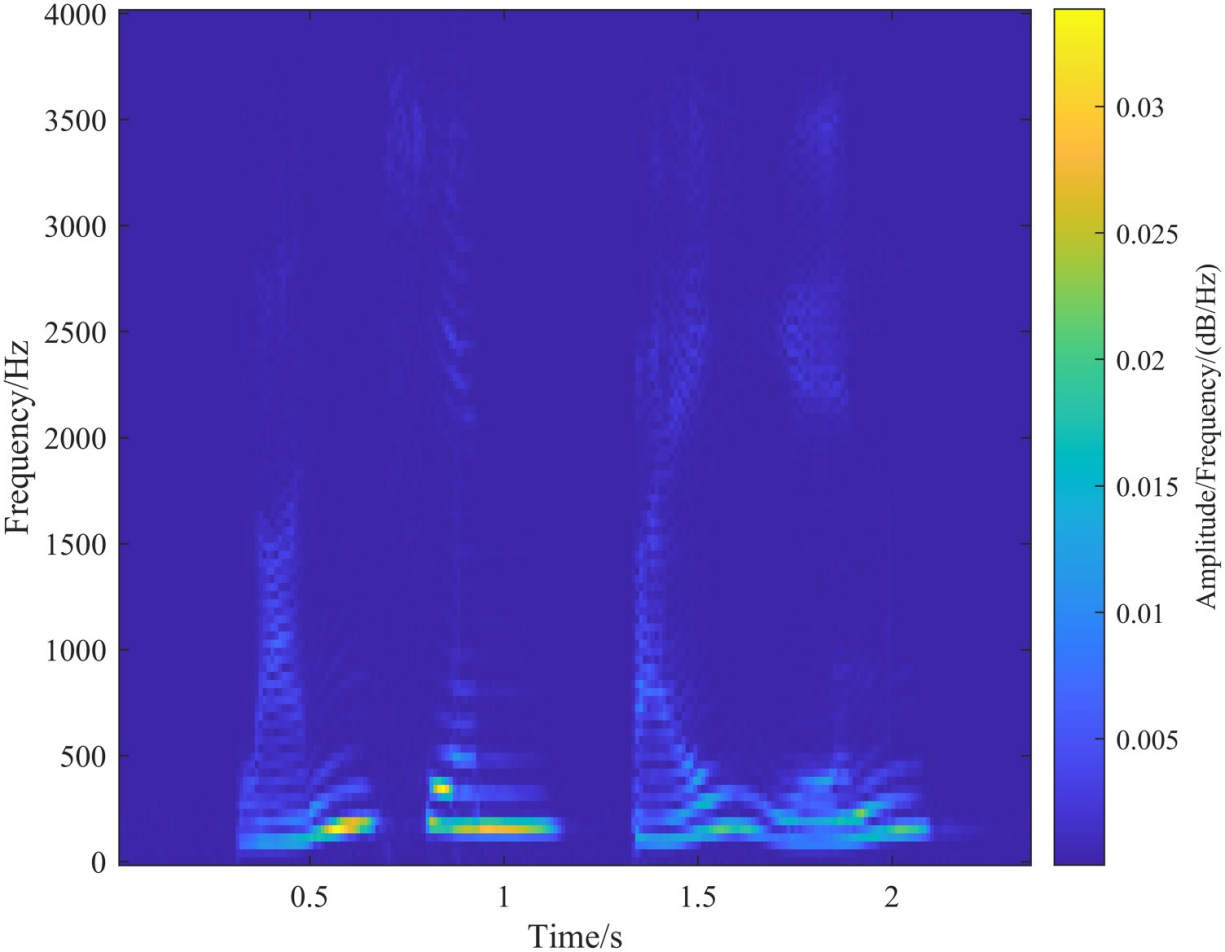
Spectrograms extracted from the generalized S-transform corresponding to the adjustment parameters λ_*G*_ = 0.05, λ_*L*_ = 0.01.

However, experiments indicate that when λ_*G*_ is set to 0.05, adjusting λ_*L*_ does not significantly affect the resolution of the generalized S-transform, as shown in Fig 9. Similarly, when λ_*L*_ is set to 0.01, the resolution remains largely unchanged, as shown in Fig 10. The spectrograms in Figs 9 and 10 closely resemble the spectrogram in Fig 8(d) when λ_*G*_ = 0.25 and λ_*L*_ = 0.01. Therefore, the generalized S-transform parameters λ_*G*_ = 0.05 and λ_*L*_ = 0.01 are selected for speech data, as depicted in Fig 11. This flexibility in adjusting the parameters λ_*G*_ and λ_*L*_ in the generalized S-transform allows for obtaining spectrograms with different resolutions, making it highly adaptable for speech signal processing.

From the S-transform results (Fig 6), it can be seen that the speech signal is S-transformed to show the distribution of the fundamental frequency, but when showing the resonance peaks, the time resolution is high, the frequency resolution is low, and the fundamental part and the second resonance peak are not finely delineated into two parts.

Upon comparing and analyzing Fig 11, it is clear that the generalized S-transform result allows for a finer distinction between the fundamental frequency and each resonance peak of the speech signal, with a marked improvement in the frequency resolution of both the fundamental and resonance peaks. The figure shows that the fundamental frequency of the speech signal lies between 100Hz and 200Hz. The signal strength at each time point is represented by the grayscale in the speech spectrogram. The curve parallel to the fundamental frequency waveform corresponds to the resonance peak information at the same time period. The speech spectrogram effectively reflects the dynamic spectral characteristics of the speech signal, providing a visual representation of the speech.

**Fig 12:**
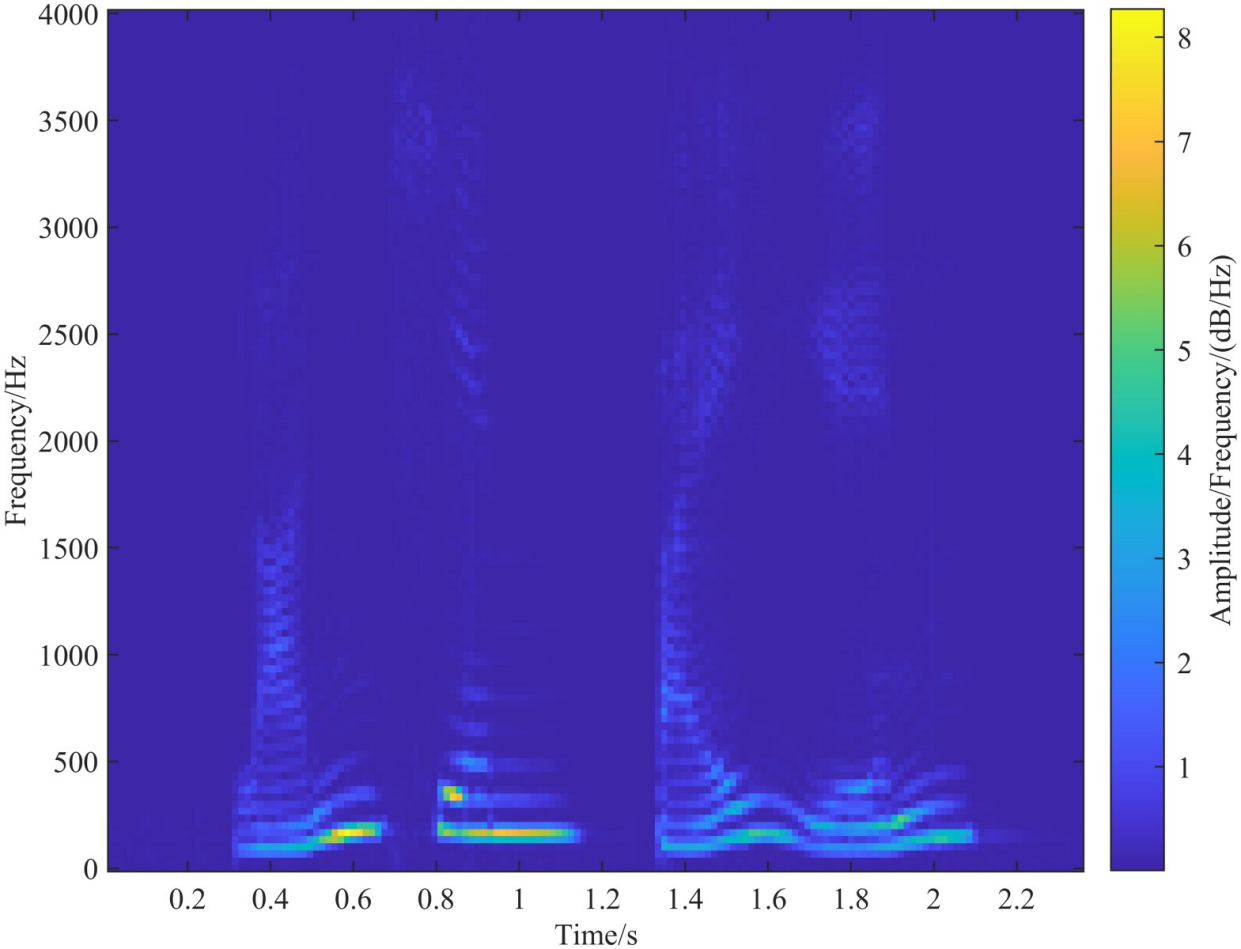
Speech spectrogram extracted from speech signal processed by short time Fourier transform method.

**Fig 13:**
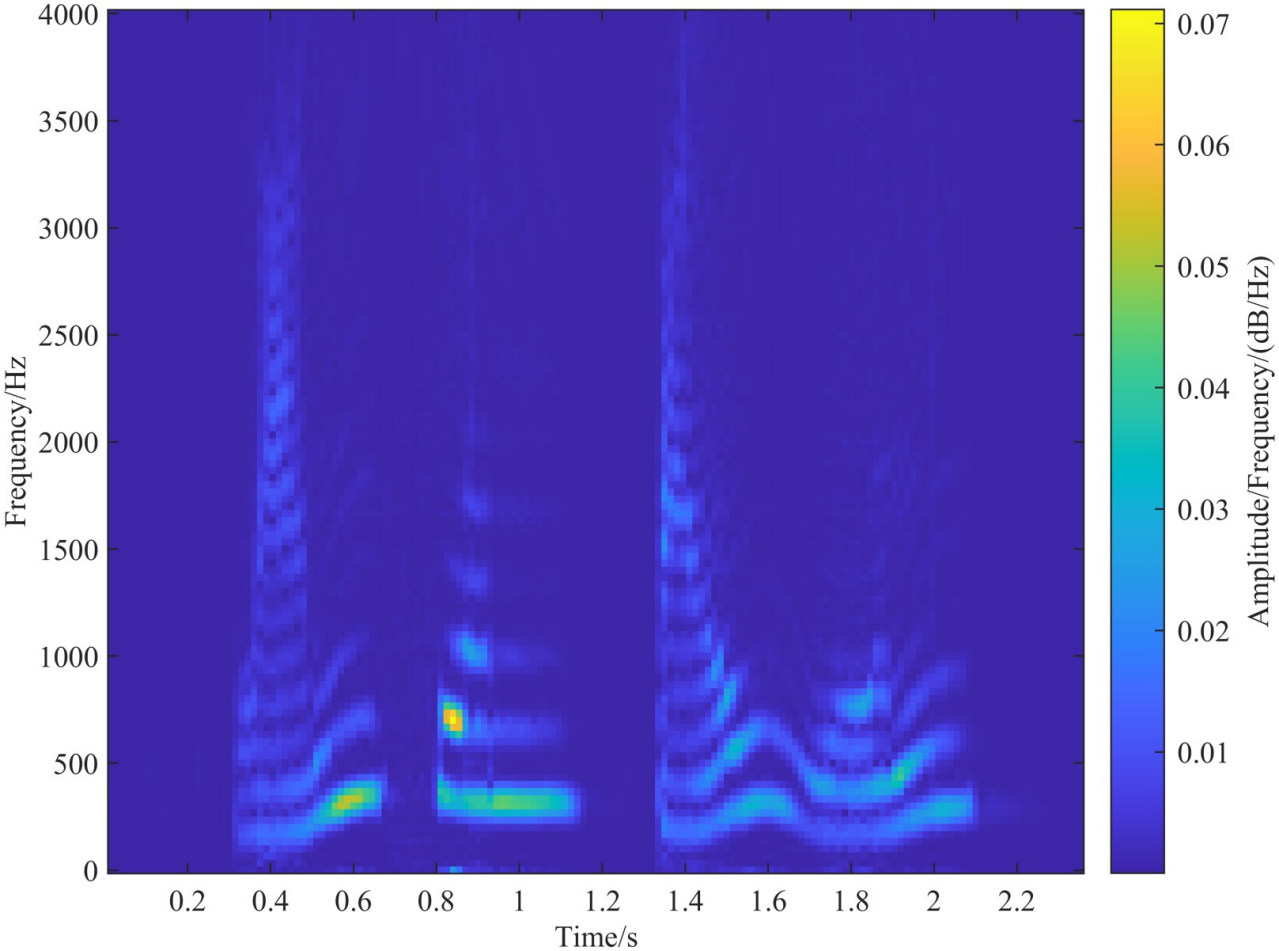
Speech spectrogram extracted from speech signal processed by wavelet transform method (Morlet mother wavelet with center frequency parameter of 50 and bandwidth parameter of 3 was selected).

Under the same experimental conditions, time-frequency analyses of the speech signal were performed using the Short-Time Fourier Transform (STFT) and the wavelet transform (using a Morlet mother wavelet with a center frequency parameter of 50 and a bandwidth parameter of 3). The resulting spectrograms are shown in Figs 12 and 13, respectively. The spectrograms obtained from the generalized S-transform in Fig 11 are similar to those from the STFT in Fig 12, with the fundamental frequency primarily distributed between 100 Hz and 200 Hz. Within the effective speech range, the patterns of speech sound distribution are consistent, and the resonance peaks are distinct and similarly distributed.

In contrast, Fig 13, which shows the spectrogram obtained via wavelet transform, demonstrates higher fundamental frequency resolution but lower time resolution. In the distribution of high-frequency resonance peaks, the time resolution is higher while the frequency resolution is lower. When compared with the fundamental frequency distribution in Fig 11, the two are similar, and the acoustic texture distribution within the effective speech is comparable, corresponding to the time-domain distribution of the speech signal in Fig 5. Comparative analysis with the spectrograms obtained from the STFT and wavelet transform indicates that the generalized S-transform is a feasible method for extracting speech signal spectrograms.

## 5 Evaluating the effectiveness of GST in acquiring speech maps

The experiment demonstrates that GST is a feasible method for extracting speech spectrograms. To validate the effectiveness of GST in obtaining accurate speech spectrograms, the inverse spectral method is employed to extract the fundamental frequency curve of speech, serving as a reference index. The effectiveness of the spectrogram is then verified against this reference. The formula for extracting the speech gene frequency curve using the inverse spectral method is as follows:

The Fourier transform of the signal x(n) is given by:

$$\begin{array}{c} X(\omega )=FT[x(n)] \end{array}$$ (16)

The process of applying a logarithm to the amplitude after performing the Fourier transform, followed by solving for the inverse Fourier transform, is known as cepstrum [2].

$$\begin{array}{c} \hat{x(n)}=FT^{-1}[ln(\left|X\left(\omega \right)\left|\right)\right] \end{array}$$ (17)

The cepstrum sequence $\hat{x(n)}$ of x(n) is the inverse Fourier transform of the logarithm of the magnitude spectrum of x(n), where FT and *FT*^−1^ denote the Fourier transform and inverse Fourier transform, respectively.

A similar method is applied to the speech signal, where the speech signal x(n) is obtained from the excitation pulse u(n) filtered by the vocal tract response v(n). The speech signal can be modeled as:

$$\begin{array}{c} x(n)=u(n)*v(n) \end{array}$$ (18)

The Fourier transform of this model is:

$$\begin{array}{c} X(\omega )=U(\omega )V(\omega ) \end{array}$$ (19)

Solving for the cepstrum yields:

$$\begin{array}{c} \hat{x(n)}=FT^{-1}ln(|U(\omega )|)+FT^{-1}ln(|V(\omega )|) \end{array}$$ (20)

**Fig 14:**
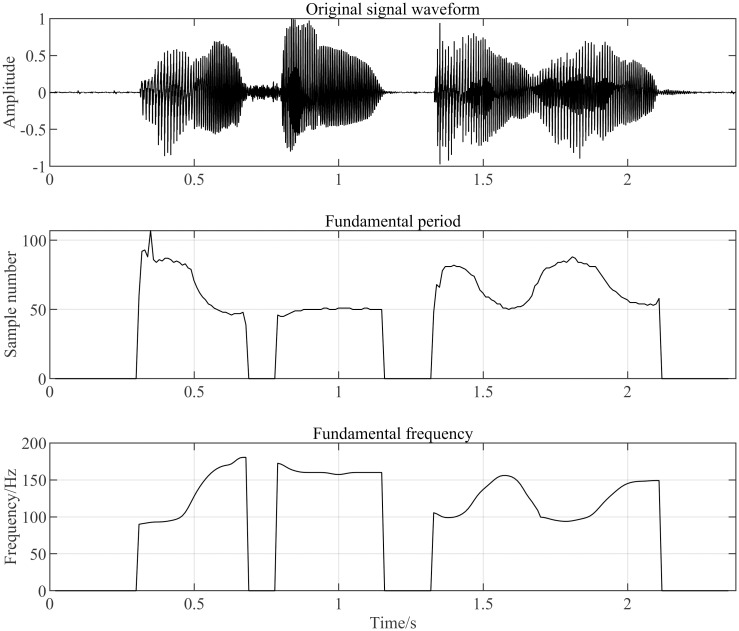
Cepstrum method for extracting the fundamental frequency curve in a speech signal, where the top graph shows the time domain distribution of the speech signal, the middle graph shows the distribution of fundamental period sampling points, and the bottom graph shows the distribution of gene frequency calculated by the sampling points of the middle graph.

**Fig 15:**
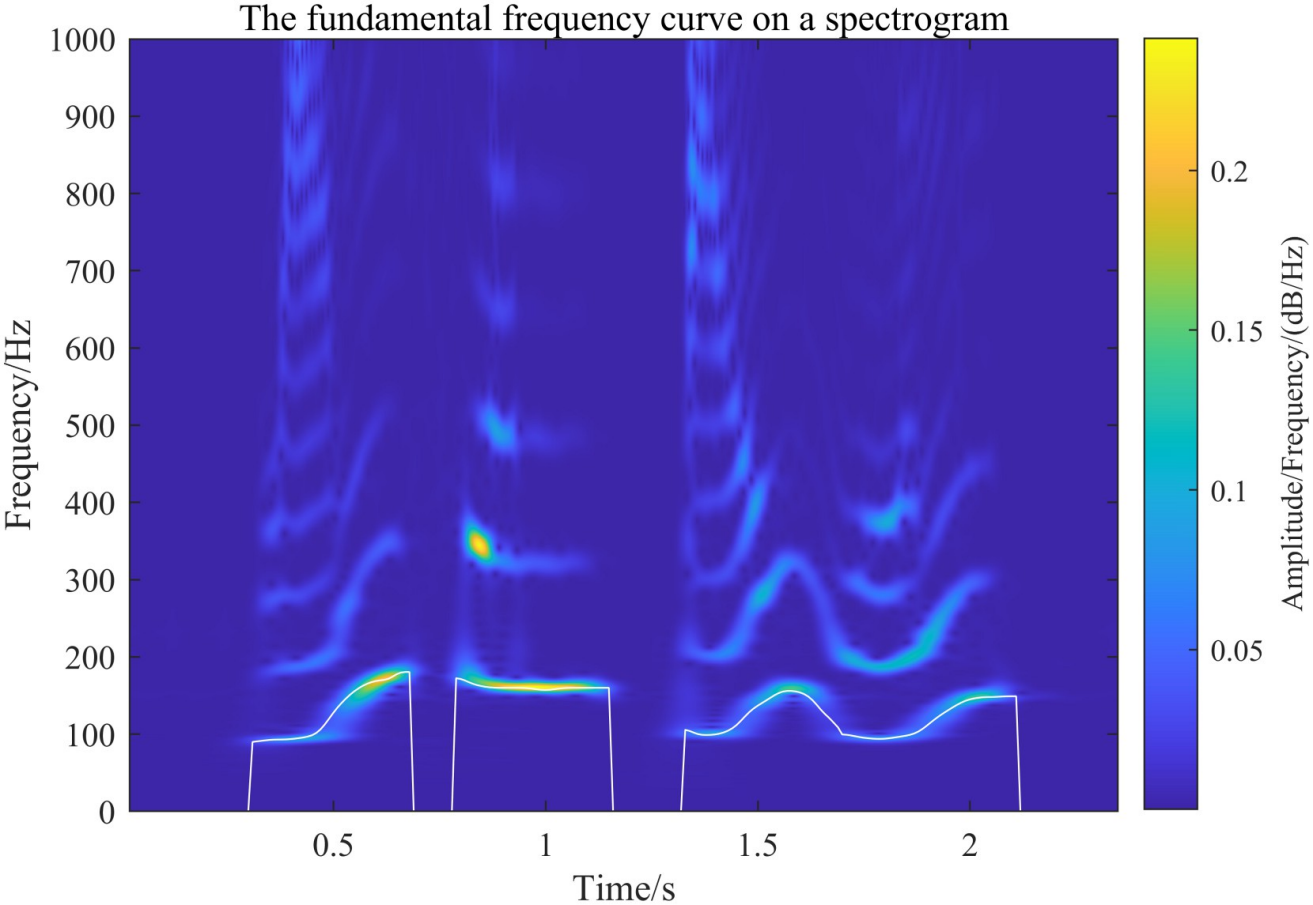
Generalized S-transform to extract the speech spectrogram and superimpose the fundamental frequency profile onto the spectrogram resultant plot.

**Fig 16:**
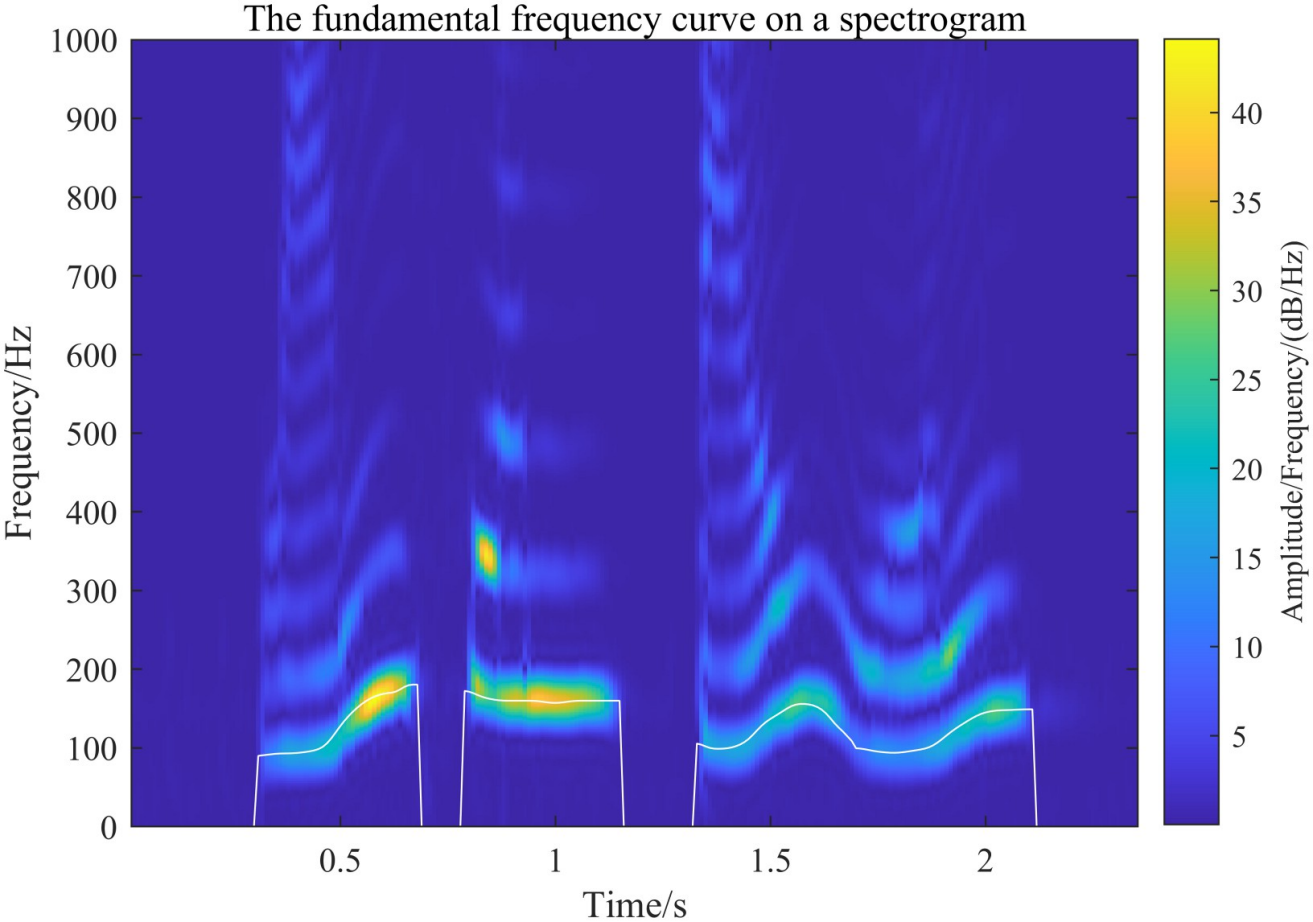
Resulting plot of STFT extracting the speech spectrogram and superimposing the fundamental frequency curve onto the spectrogram.

**Fig 17:**
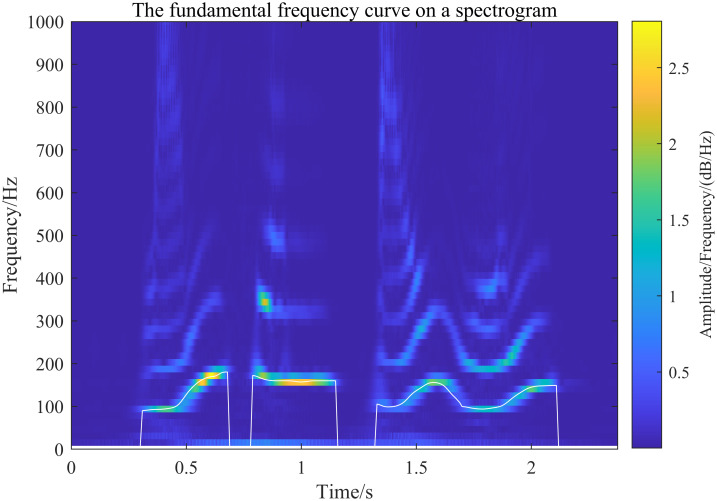
Wavelet transform to extract the speech spectrogram and superimpose the fundamental frequency curve onto the spectrogram resultant plot.

From Eqs (16) to (20), it is evident that the cepstrum of the acoustic pulse *FT*^−1^*ln*(|*U*(*ω*)|) and the cepstrum of the vocal tract response *FT*^−1^*ln*(|*V*(*ω*)|) can be separated by the cepstral algorithm. This separation allows for the recovery of the excitation signal u(n) from the cepstrum domain *FT*^−1^*ln*(|*U*(*ω*)|). The distribution of the fundamental frequency curve in the final speech signal is shown in Fig 14 below:

The fundamental frequency curve is obtained using the inverse spectral method and mapped onto the spectrogram. To verify the effectiveness of the generalized S-transform (GST) in obtaining the spectrogram, it is compared with the STFT and wavelet transform algorithms, using the fundamental frequency curve as a reference. In this comparison, the STFT uses a fixed Hamming window with a width of 30 ms, while the wavelet transform employs the Morlet wavelet with a center frequency parameter of 3 and a bandwidth parameter of 3. The results of each algorithm are shown in Figs 15 to 17 below.

Fig 15 shows the spectrogram of the fundamental frequency obtained by GST (with selected parameter values of λ_*G*_ = 0.2 and λ_*L*_ = 0.1, providing high resolution of the fundamental frequency). Based on the alignment of the fundamental frequency curves and the spectrogram’s distribution, it can be confirmed that the speech spectrogram extracted by the generalized S-transform proposed in this paper is effective. Figs 16 and 17 illustrate the spectrograms obtained by the STFT and wavelet transform, respectively. The comparison of these figures, under the same fundamental frequency distribution curve, shows that the distribution of the fundamental frequency is consistent across the spectrograms extracted by all three methods. This verifies that the speech spectrograms extracted by the generalized S-transform method proposed in this paper are both feasible and effective for speech signals.

## 6 Conclusion

In this paper, we introduce a low-pass filter to the time window function based on the S-transform and incorporate two adjustment parameters to control the time and frequency resolution of the generalized S-transform. By selecting appropriate adjustment parameters, the generalized S-transform can be effectively tailored for processing speech signals. Experimental comparisons demonstrate that the generalized S-transform offers high flexibility and feasibility in extracting speech spectrograms. In the future, our research group will explore the application of the generalized S-transform in noisy speech and the train with large datasets.

## Supporting information

S1 Video The content of the speech. (WAV)
